# Influence of anxious attachment on the relationship between primary emotions and substance-related addictive behaviors

**DOI:** 10.3389/fpubh.2024.1380539

**Published:** 2024-06-17

**Authors:** Jürgen Fuchshuber, Deborah Andres, Theresa Prandstätter, Lisa Roithmeier, Beate Schmautz, Andreas Schwerdtfeger, Human-Friedrich Unterrainer

**Affiliations:** ^1^Center for Integrative Addiction Research (CIAR), Grüner Kreis Society, Vienna, Austria; ^2^Department of Psychoanalysis and Psychotherapy, Medical University of Vienna, Vienna, Austria; ^3^Comprehensive Center for Clinical Neurosciences and Mental Health, Medical University Vienna, Vienna, Austria; ^4^Institute of Psychology, University of Graz, Graz, Austria; ^5^University Clinic for Psychiatry and Psychotherapeutic Medicine, Medical University Graz, Graz, Austria; ^6^Department of Religious Studies, University of Vienna, Vienna, Austria; ^7^Faculty of Psychotherapy Science, Sigmund Freud University, Vienna, Austria

**Keywords:** primary emotions, substance use, attachment anxiety, addictive behavior, structural equation modeling, mediation

## Abstract

**Objectives:**

To date there is no universally accepted model that describes the development of substance related addictive behavior. In order to address this gap, the study sought to examine whether the association between primary emotions and the inclination toward addictive behavior is mediated by an anxious attachment style.

**Methods:**

The total sample consisted of 900 German speaking non-clinical adults (age: *M* = 27; *SD* = 9.60; 71.6% female). Structural Equation Modeling (SEM) was applied to examine the connection between the primary emotions (SADNESS and ANGER), and the latent variables attachment anxiety and symptoms of addictive behavior.

**Results:**

Substance use symptomatology was correlated with higher attachment anxiety (*r* = 0.15), SADNESS (*r* = 0.15), and ANGER (*r* = 0.11). The effect of SADNESS on addictive behavior is mediated by attachment anxiety (*p* < 0.01) whereas ANGER had a direct effect on addictive behavior (*p* < 0.01). The final SEM explains 4% of the variance of addictive behaviors and 22% of attachment anxiety.

**Conclusion:**

Our findings suggest that both SADNESS and ANGER, along with attachment anxiety, are dispositions that contribute to the risk of engaging in addictive behavior. However, while ANGER directly influences addictive behavior, SADNESS acts through its impact on attachment anxiety.

## Introduction

Substance use disorder (SUD) is defined as a pathological, chronic, and compulsive urge to consume psychoactive substances although they are hazardous to oneself and others (1). The 11th revision of the International Classification of Disease [ICD-11; (2)] differentiates “Disorders due to substance use or addictive behaviors” (6C4) between “hazardous substance use,” “episodes of harmful use,” “harmful substance use,” and “substance dependence.” The diagnosis is based on the presence of the following criteria: (1) Craving or compulsion to use the substance; (2) difficulties in controlling substance use; (3) persistent substance use despite harmful consequences; (4) prioritization of substance use over other activities and responsibilities; (5) increased tolerance; and (6) withdrawal symptoms.

Addictive behaviors toward substance use such as the consumption of alcohol, nicotine, cannabis use and others, are widespread among the general population (3) and are associated with serious public health problems. Correspondingly, the World Drug Report (3) notes that 284 million people worldwide are currently affected by substance use and dependence. Younger generations typically report higher levels of substance use than adults. Moreover, substance use levels among young people is higher today than in previous generations. In addition, SUDs show significant comorbidities with a variety of psychiatric disorders (4). SUDs are considerably related to depression, anxiety disorder, bipolar and emotional dysregulation disorder (4, 5). In terms of the impact of emotions on the pathogenesis of addiction, recent advances in Affective Neuroscience (AN) propose specific dysregulations within primary emotions. According to Panksepp(6) primary emotions are a subcortically rooted affective systems that are thought to be evolutionarily innate, serving as the primary motivational system of behavior and are universal across cultures. The proposed seven basic emotional systems are evolutionary tools for survival and fitness in mammalian species and are located in the subcortical brain.

These primary emotions systems are called SEEKING, CARE, LUST, ANGER, PLAY, FEAR, PANIC/GRIEF (or SADNESS) (7). Based on their valence they can be roughly distinguished into a positive and a negative group (8). The positive group encompasses SEEKING, PLAY, CARE, and LUST, which collectively reside on the pleasurable end of the emotional spectrum. They play integral roles in accomplishing diverse life objectives, such as engaging with the external world (SEEKING), achieving reproduction (LUST), nurturing offspring (CARE) or fostering social connections (PLAY). In contrast FEAR, ANGER and SADNESS represent are unpleasurable primary emotions. The evolutionary use of these emotions is, e.g., the avoidance of harm, overcoming of obstacles or aversion of loss. The negative emotions can be understood as an innate warning system (9).

The Affective Neuroscience Personality Scales (ANPS) were developed by Davis et al. (10) to measure primary emotions dispositions in individuals. Furthermore, Barrett et al. (11) developed a short version of the ANPS, defined as the BANPS. Most recently, Fuchshuber et al. (12) developed The German Version of the Brief Affective Neuroscience Personality Scales including a LUST scale (BANPS–GL). Regarding the impact of primary emotions on the pathogenesis of SUDs recent studies emphasize dysregulations especially within the SEEKING, the SADNESS and the ANGER systems (13, 14).

The SEEKING network conveys positive feelings of curiosity and anticipation and is consistent with Berridge’s concept of “wanting” (15). In terms of the SEEKING system, addictive behavior is thought to be characterized by pathological alterations occurring within the SEEKING/mesolimbic dopamine system. Subsequently, addictive behavior is mediated by obsessive behaviors, maintained by wanting/SEEKING circuits in the case of substance use, as well as consummatory-PLEASURE reactions, particularly in the case of opioid abuse (14). In turn, addictive behavior may eventually lead to the exhaustion of resources for reward seeking. This in turn causes a feeling of dysphoria that can only be relieved temporarily by substance use. However, this promotes a negative effect that perpetuates the addictive cycle. Finally, the SEEKING network is increasingly activated in relation to substance-related appetitive memories, substance use, and the drive to relieve negative affective states (16–18).

The ANGER system is described by the characteristic of feeling easily irritated, frustrated and aggressive, as well as the experience of being angry as a result of frustrations. The hypothesis that anger and aggression is related to addictive behavior is based on object relations theory, which views (auto-) aggressive behavior as an etiological factor in substance use (19, 20). In the tradition of Glover (21), who understood drug use as attack against hated internalized objects, which the patients perceives as a hostile foreign body, Rosenfeld argues that addiction involves a defensive manic formation centered around idealizing, identification with ideal objects, denial of persecutory fears and depressive anxiety. In addition to its defensive function, drug intake is conceptualized as an identification with sadistic and destructive objects, which persecute the good representations of objects and the self in order to achieve omnipotent control of them. This enables the acting out of sadistic impulses without concerns, feelings of guilt or control by the super-ego. Finally, according to Rosenfeld, drug use might also serve a masochistic function, as the drug symbolizes a dead or damaged object which the patient feels obligated to incorporate and ultimately identify with, due to intense and unbearable feelings of guilt. Recent results by Unterrainer et al. (22) and Fuchshuber et al. (23) resonate with these considerations on a psychometric level: Unterrainer et al. (22) were able to show increased SADNESS, FEAR and ANGER in substance use disorder patients. Employing a path analytical approach, Fuchshuber et al. (23) found associations between substance use, ANGER and SADNESS in a non-clinical sample.

The SADNESS system is triggered by the loss of a beloved object and consequently produces painful feelings of separation anxiety/distress and loneliness (24). The SADNESS system, which is also called the PANIC/GRIEF system, based on the panic phase of separation distress, e.g., when a child has lost the contact with his caregivers, but also due to his relation to “panic” attacks. This network is also activated in a similar way when a social/romantic relationship ends. When attachments are interrupted by loss or separation, the person feels “bad” in a special way. This particular type of social pain is referred to as “separation distress.” Neurochemically, this circuit is predominantly controlled by the endogenous opioid system (25–27). Endogenous mu and delta opioid receptor ligands (like enkephalins and endorphins) deactivate the PANIC/GRIEF system, while kappa-opioid ligands (like dynorphins) increase its activity (28). On this background, SUDs – and specifically SUDs involving the use of opioids – might be seen as a dysfunctional attempt to (self-) regulate overwhelming feelings of loss, sadness, grief, panic and isolation mediated by an overactive PANIC/GRIEF system.

Furthermore, the predominantly primary emotion systems, are considered as connected to secondary order processes, which includes attachment patterns in mammals (29). The development of attachment bonds, which is especially driven by the SADNESS system, and addictive behavior has strong similarities (30, 31). Those similarities shared by attachment and addiction involve social bonding and drug dependence, estrangement and drug tolerance as well as separation distress and drug withdrawal. As a result, addiction is frequently described as a deranged type of attachment (32). Attachment Theory, originally posited by Bowlby (33) and subsequently refined by Ainsworth (34), postulates that the development of forms of psychopathology in adolescence and young adulthood may be due, at least in part, to dysfunctional interactions with attachment figures and influences affect regulations. According to this theory, these are securely attached and two insecurely attached attachment patterns: Attachment anxiety – an overactive pattern – is defined by the fear of interpersonal rejection and the excessive search for closeness and recognition by others, combined with low self-confidence. On the contrary, attachment avoidance – a deactivation pattern – is associated with fears of closeness and interpersonal dependence (35).

There is a growing amount of empirical evidence linking adult attachment styles and addictive disorders (36, 37). A meta-analysis of 34 studies (*N* = 56,721) found significant associations linking insecure attachment and substance use (37). Insecure attachment style was found to predict the development of substance use problems and the association remained the same regardless of the kind of substance (e.g., alcohol and marijuana).

Regarding the association between addictive behavior and insecure attachment style, associations are found particularly with anxious attachment style. It is hypothesized that people who report stronger anxious attachment may turn to addictive behaviors to cope with emotional distress, whereas those with an avoidant style may not experience such emotional distress because of their strategies to deactivate emotions. Schindler et al. (38) found associations between attachment anxiety and substance use disorder as well as Unterrainer et al. (22) and Liese et al. (39).

A meta-analysis of 100 studies (*N* = 20,350) supporting the role of attachment and emotional dysregulation found that insecure attachment is consistently linked to lower emotion regulation skills than secure attachment (40). In fact, emotion dysregulation has been hypothesized to mediate the link between anxious (but not avoidant) attachment and alcohol problems (41).

Of note, recent evidence regarding the genetic underpinnings of adult attachment highlight not only candidate genes related to oxytocin pathways and brain-derived neurotrophic factor (42) but also the involvement of the mu-opioid receptor gene (43), especially in regards to avoidant attachment. The shared neurobiological underpinnings linked to the endogenous opioid system might in part explain previously observed associations between adult attachment and SADNESS dispositions (44).

### Study aims

To further investigate the interactions between primary emotions, attachment anxiety and addictive behavior, this study examines whether the relationship between primary emotions and the tendency toward addictive behavior is mediated by an anxious attachment style. To our knowledge, no study has tested whether attachment anxiety mediates the relationship between specific primary emotions and addictive behavior.

In line with previous research we hypothesized that increased negative primary emotions (FEAR, ANGER, and SADNESS) and attachment anxiety are associated with addictive behavior. To assess the extent of how attachment anxiety mediates the association between primary emotions and addictive behavior, this study applied the structural equation modeling technique, which has the advantage of being able to estimate the relationship of multiple concepts simultaneously.

## Materials and methods

### Sample and procedure

The total sample consisted of 900 non-clinical and German-speaking adults (gender: 71.6% female; age: 18–73 years, *M* = 27.74, *SD* = 79.60). No possible exclusion criteria have been mentioned in the literature so far. Since language is significant for comprehension, the criteria for participation were an age of over 18 years and fluency in German. From the original sample of 1,566 participants, 659 who had not completed the survey were excluded. In addition, two participants were excluded who had not consented to the processing of their data and further 5 subjects who had provided incorrect information. The participants were recruited via public announcements at the University of Graz, a student’s mail distribution list and social networks such as Instagram, Studio, and Facebook. The questionnaires were collected through the online-survey platform LimeSurvey^©^. Informed consent was obtained from all participants prior to answering the questions. The survey contained multiple different sociodemographic questions (e.g., age, gender, education, and psychiatric diagnosis) and standardized test procedures. No compensation was offered. Participants were completely anonymous at all times. The study was carried out in accordance with the Declaration of Helsinki. The Ethics Committee of the University of Graz, Austria granted ethical approval. Study participants were recruited from August 2022 to January 2023.

### Psychometric assessment

After obtaining informed consent, the participants were given a sociodemographic questionnaire in which all personal data relevant to the study were collected. The demographic questionnaire consisted of questions on age, gender, marital status, education level and field of study, current occupation or training, current presence of psychiatric disorders, medication as well as country of origin and language skills.

#### Primary emotions

The *German Version of the Brief Affective Neuroscience Personality Scales including a LUST Scale* [BANPS–GL; (12)] is a self-report questionnaire which, with an additional LUST scale (45), covering all seven primary emotions developed by Panksepp (24). The Brief–Affective Neuroscience Personality Scales [BANPS; (11)] represents a shortened version of the Affective Neuroscience Personality Scales (10). Hence, this questionnaire includes the subscales “PLAY,” “CARE,” “SEEKING,” “ANGER,” “FEAR,” and “SADNESS” as well as an additional scale for the dimension LUST. It consists of 33 items and is rated on a 5-point scale from (1) strongly disagree to (5) strongly agree. The BANPS-GL exhibits acceptable to good internal consistencies ranging from Cronbach’s *α* = 0.69 (CARE) to *α* = 0.85 (SADNESS) (12).

#### Addictive behavior

The *World Health Organization’s Alcohol, Smoking and Substance Involvement Screening Test* [ASSIST; (46)] is a standardized interview to ascertain psychoactive substance use and substance use related problems. For the intent of this online study, the ASSIST was adapted as a self-report questionnaire. The WHO ASSIST ascertains lifetime use and symptoms of abuse of 10 substance groups, which include tobacco, alcohol, and cannabis, cocaine, amphetamines, inhalants, sedatives, hallucinogens, opioids, and “other drugs.” Symptoms of drug use are assessed on a 7-point Likert scale ranging from “never” (0) to “daily or almost daily” (6) for questions 2–5, which measure “frequency of drug use,” “craving for the drug,” and “problems” (social, financial or health) resulting from drug use and “Disappointed expectations.” The questions 6, 7, and 8 are scored on a 3-point scale (0 = “no, never”; 3 = “yes, but not in the last 3 months”; 6 = “yes, in the last 3 months”) and refer to “concerns expressed by relatives or friends,” “failed attempts to cut down on drug use,” and “drug injection.” The ASSIST can assess various types of substance involvement. For the purpose of this study, we calculated a total score for each symptom class regarding substance use (“Frequency,” “Craving,” “Problems,” “Failed expectations,” “Concerns,” and “Failed attempts to cut on use”) by summing the drug-specific symptom scores. Subsequently, we calculated an overall Global Substance Use Risk Pathology score (WHO Global Substance Pathology) for this study. This scale showed excellent internal consistency with a Cronbach’s alpha = 0.94. Subsequently, we integrated the subscales into the structural equation model as indicators for the latent variable “addictive behavior.” The internal consistencies for the subscales implemented as indicators were generally acceptable ranging from *α* = 0.68 to *α* = 0.81.

#### Attachment

The abbreviated version of the *Experiences in Close Relationships-Revised* [ECR-RD8; (47)] questionnaire is an established self-report tool to assess attachment insecurity in relation to anxiety and avoidance behavior. The questionnaire contains eight items rated on a Likert scale from “strongly disagree” (1) to “strongly agree” (7). The short version achieves excellent reliability in its German version. The internal consistency, calculated according to Cronbachs Alpha, was *α* = 0.79 for anxiety and *α* = 0.85 for avoidance.

### Statistical analyses

The statistical analysis was conducted via SPSS 29.0 and the Structural Equation Modeling (SEM) was conducted with AMOS 29. SPSS was used for data management, descriptive statistics and bivariate correlations. We performed Pearson product–moment correlations to test bivariate associations. All *p-*values refer to two-tailed tests. Correlations and effect sizes are interpreted following Cohen (48) with *r* > 0.1 small, *r* > 0.3 medium, and *r* > 0.5 large effect. The theoretically developed mediation model was operationalized using a SEM and tested with maximum likelihood estimator (using bootstrapping with 2,000 samples). The main analysis was a structural equation model of the effects of primary emotions SADNESS and ANGER on addictive behavior, with attachment anxiety as a mediator. First, the data were fitted to an initial structural equation model.

After the first initial model was fitted, a pruning strategy was used to remove the non-significant paths. Goodness of fit was evaluated using maximum likelihood estimation in AMOS. To assign a metric to the variables, the one path coefficient for each latent variable was restricted to the value of one. To test for mediation and indirect effects, a bootstrap was performed with a bias-corrected confidence interval of 95% and 2,000 bootstrap samples. Model fit was evaluated according to Kline (49) by the following indices as markers for an acceptable model fit: (a) TLI (Tucker–Lewis Index) and CFI (Comparative Fit Index) >0.90; RMSEA (the square root error of approximation) ≤0.08 and the upper bound of the 90% confidence interval < 1. The Akaike information criterion (AIC) was used to compare the competing models. Both model will be controlled for sex-effects.

## Results

### Sample characteristics

The descriptive sample characteristics are detailed in Table 1. The total sample consisted of 900 German-speaking non-clinical adults with a mean age of 27.74 years and an age range of 18 to 73 years (*SD* = 9.60). From all participants included in the study 644 were females (71.6%), A total of 372 (41.2%) indicated a university degree as the highest educational qualification, 399 (44.3%) a general qualification for university entrance, 30 (3.3%) a high school degree and 98 (10.9%) participants reported a completed apprenticeship as highest educational level. Most participants were from German-speaking countries (German, Austrian, or Swiss nationality; *N* = 833; 92.6%), whereas 67 (7.4%) had a different nationality.

**Table 1:** Sample characteristics.

Sample	
Overall	*N* = 900
Gender	*N* = 644 Female (71.6%)
	*N* = 256 Male (28.4%)
Age	*M* = 27.72 (*SD* = 9.60 years)
Nationality	*N* = 833 AT, DE, CH (92.6%)
	*N* = 67 other (7.4%)
Highest educational degree	*N* = 372 University degree (41.2%)
	*N* = 399 General qualification (44.3%)
	*N* = 30 High school degree (3.3%)
	*N* = 89 Completed apprenticeship (10.9%)
Current occupation	*N* = 533 University students (59.2%)
	*N* = 357 Employed (39.7%)
	*N* = 144 Side Job (16%)
	*N* = 60 Self-employed (6.7%)
	*N* = 30 In school (3.3%)
	*N* = 25 Unemployed (2.8%)
	*N* = 17 In education (1.9%)
	*N* = 15 In pension (1.7%)
	*N* = 8 Maternity leave (0.9%)
Relationship status	*N* = 432 Single (48%)
	*N* = 332 In a relationship (36.9%)
	*N* = 107 Married (11.9%)
	*N* = 24 Divorced (2.7%)
	*N* = 3 Widowed (0.3%)
Diagnosed psychiatric disorder	*N* = 119 Yes (13.2%)
	*N* = 781 No (86.8%)
Regular medicament intake	*N* = 198 Yes (22%)
	*N* = 702 No (78%)

AT = Austria, DE = Germany, CH = Switzerland.

Regarding the current occupation of participants, 30 (3.3%) were in school, 17 (1.9%) were in education, 533 (59.2%) were university students, 357 (39.7%) were employed, 60 (6.7%) were self-employed, 8 (0.9%) persons were in maternity leave, 15 (1.7%) were retired, 25 (2.8%) were unemployed and 144 (16%) had a side job. As it was possible to give multiple answers, the percentages do not add up to a 100%. Regarding the current relationship status 432 (48%) of the participants were single, 332 (36.9%) were in a relationship, 107 (11.9%) were married, 24 (2.7%) were divorced and 3 (0.3%) were widowed. Concerning the psychiatric disorder 119 (13.2%) of the surveyed said, that they were at one point of their live diagnosed with a psychiatric disorder. Finally, 198 (22%) of the people said, that they take medicaments on a regular basis.

### Correlations

The bivariate correlations (Table 2) between the analyzed variables revealed that Substance use symptomatology was significantly positively correlated with higher levels of attachment anxiety (*r* = 0.15, *p* < 0.001) and attachment insecurity (*r* = 0.10, *p* < 0.01) but did not correlate with attachment avoidance (*r* = −0.01, ns.). Furthermore, substance use behavior correlated with higher levels of primary emotions SADNESS (*r* = 0.15, *p* < 0.001) and ANGER (*r* = 0.11, *p* < 0.001) but did not correlate with primary emotions PLAY (*r* = 0.02, ns.), CARE (*r* = −0.01, ns.), SEEKING (*r* = 0.01, ns.), FEAR (*r* = 0.03, ns.), and LUST (*r* = 0.00, ns.).

**Table 2:** Descriptive statistics and zero-order correlations among variables for the measurement model.

		1	2	3	4	5	6	7	8	9	10	11	12	13	14	15	16	17	18
1.	BANPS PLAY	–																	
2.	BANPS CARE	0.43^***^	–																
3.	BANPS SEEKING	0.28^***^	0.22^***^	–															
4.	BANPS LUST	0.38^***^	0.39^***^	0.20^***^	–														
5.	BANPS ANGER	−0.08^*^	−0.15^***^	−0.11^***^	−0.07^*^	–													
6.	BANPS FEAR	−0.15^***^	0.03	−0.05	−0.27^***^	0.26^***^	–												
7.	BANPS SADNESS	−0.23^***^	−0.06	−0.10^**^	−0.35^***^	0.34^***^	0.68^***^	–											
8.	Attachment insecurity	−0.25^***^	−0.26^***^	−0.19^***^	−0.36^***^	0.25^***^	0.23^***^	0.44^***^	–										
9.	Attachment anxiety	−0.09^*^	0.02	−0.08^*^	−0.19^***^	0.18^***^	0.29^***^	0.42^***^	0.81^***^	–									
10.	Attachment avoidance	−0.31^***^	−0.46^***^	−0.22^***^	−0.39^***^	0.21^***^	0.06	0.26^***^	0.76^***^	0.23^***^	–								
11.	WHO Substance Global	0.02	−0.01	0.01	0.01	0.10^**^	0.03	0.11^***^	0.10^**^	0.15^***^	0.00	–							
12.	WHO Frequency	0.09^**^	0.04	0.03	0.11^***^	0.05	−0.04	0.05	0.03	0.07^*^	−0.03	0.81^***^	–						
13.	WHO Craving	0.03	0.01	0.01	0.02	0.14^***^	0.08^*^	0.15^***^	0.11^**^	0.17^***^	−0.01	0.84^***^	0.71^***^	–					
14.	WHO Problems	−0.02	−0.02	−0.05	−0.03	0.09^**^	0.02	0.09^*^	0.09^**^	0.14^***^	0.00	0.73^***^	0.44^***^	0.48^***^	–				
15.	WHO Failed expectations	0.00	−0.03	−0.03	0.00	0.05	0.03	0.06	0.08^*^	0.12^***^	−0.01	0.70^***^	0.44^***^	0.47^***^	0.61^***^	–			
16.	WHO Concerns	0.02	0.00	0.04	−0.02	0.06	0.01	0.10^**^	0.06	0.10^**^	0.00	0.78^***^	0.52^***^	0.56^***^	0.49^***^	0.45^***^	–		
17.	WHO Failed attempts	−0.03	−0.05	−0.01	−0.06	0.06	0.01	0.06	0.10^**^	0.11^**^	0.04	0.78^***^	0.53^***^	0.54^***^	0.58^***^	0.53^***^	0.51^***^	–	
18.	Sex	0.05	−0.11^**^	0.00	0.21^***^	−0.01	−0.29^***^	−0.19^***^	−0.01	−0.09^**^	0.08^*^	0.05	0.12^***^	0.00	0.01	0.05	0.02	0.01	–
	*M* or *N*	22.02	15.15	23.24	18.83	15.32	16.96	17.39	23.71	12.99	10.73	17.78	6.06	4.86	1.11	1.40	2.45	1.89	1.28
	*SD* or %	4.06	2.98	3.58	3.95	4.93	4.33	5.21	8.80	5.90	5.32	20.88	4.85	5.84	3.22	3.36	5.24	4.18	0.45
	Cronbach’s Alpha	0.77	0.69	0.73	0.78	0.80	0.84	0.86	0.78	0.79	0.84	0.94	0.68	0.71	0.81	0.77	0.81	0.76	71.6

*N* = 900; ^***^p < 0.001, ^**^p < 0.01, ^*^p < 0.05; sex was coded as 1 = female; 2 = male.

ANGER was significantly correlated with the WHO-Subscale “Craving” (*r* = 0.14, *p* < 0.001.) and “Problems” (*r* = 0.09, *p* < 0.01), as well as with attachment anxiety (*r* = 0.18; *p* < 0.001).

SADNESS was significantly correlated with the WHO-Subscale “Craving” (*r* = 0.15; *p* < 0.001), “Problems” (*r* = 0.09; *p* < 0.01), and “Concerns” (*r* = 0.10; *p* < 0.001), as well as with attachment anxiety (*r* = 0.42; *p* < 0.001).

With regard to sex differences, we observed higher CARE (*r* = −0.11; *p* < 0.001), FEAR (*r* = −0.29; *p* < 0.001), and SADNESS (*r* = −0.19; *p* < 0.001) dispositions, as well as slightly more attachment anxiety (*r* = −0.09; *p* < 0.001) in females. Male sex was correlated with increased LUST (*r* = 0.21; *p* < 0.001), avoidance (*r* = −0.08; *p* < 0.01) and frequency of substance use (*r* = 0.12; *p* < 0.001).

As demonstrated in Table 3, all latent variables included into the SEM had significant correlations among themselves (*p* < 0.001).

**Table 3:** Correlations among latent variables for the measurement model.

		1	2	3	4
1.	BANPS SADNESS	–			
2.	BANPS ANGER	0.34^***^	–		
3.	Attachment anxiety	0.42^***^	0.18^***^	–	
4.	WHO Substance Global	0.11^***^	0.10^***^	0.15^***^	–

*N* = 900; ^***^p < 0.001.

### Structural equation model

**Figure 1:**
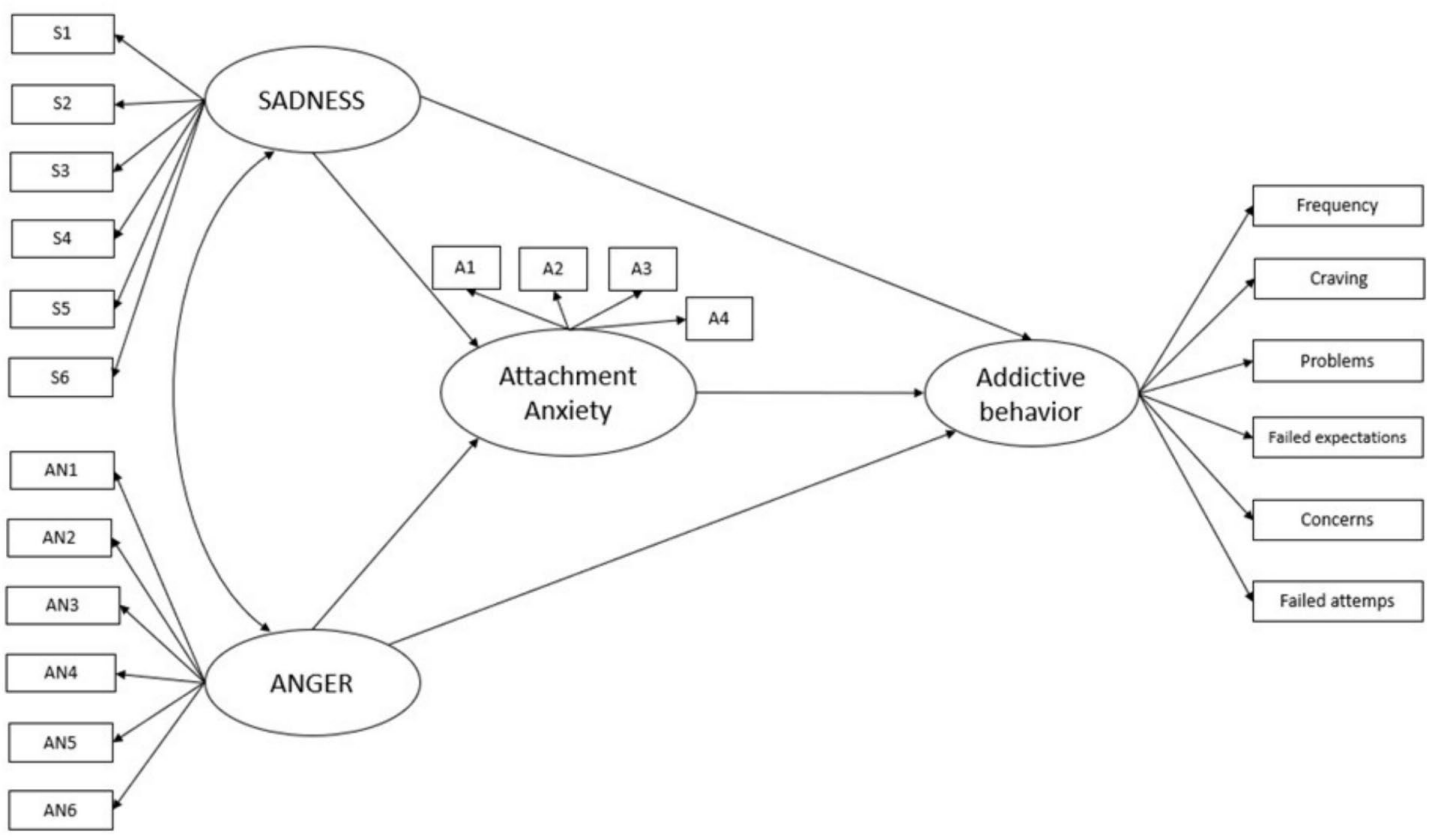
Depiction of the theoretical model. The effects of SADNESS and ANGER on addictive behavior are mediated by attachment anxiety.

The structural equation model consisted of five latent variables: The three primary emotions SADNESS, FEAR, and ANGER, Attachment Anxiety and Addictive Behaviors. All indicators loaded significantly onto their corresponding latent factors (*p* < 0.001). An initial model proposed direct effects from the correlated primary emotions SADNESS, FEAR, and ANGER to attachment anxiety and addictive behavior. However, in this model FEAR did not significantly contribute to the prediction of the outcome variable due to its high overlap with SADNESS and was subsequently deleted. This resulted in a second model which is displayed in Figure 1.

**Figure 2:**
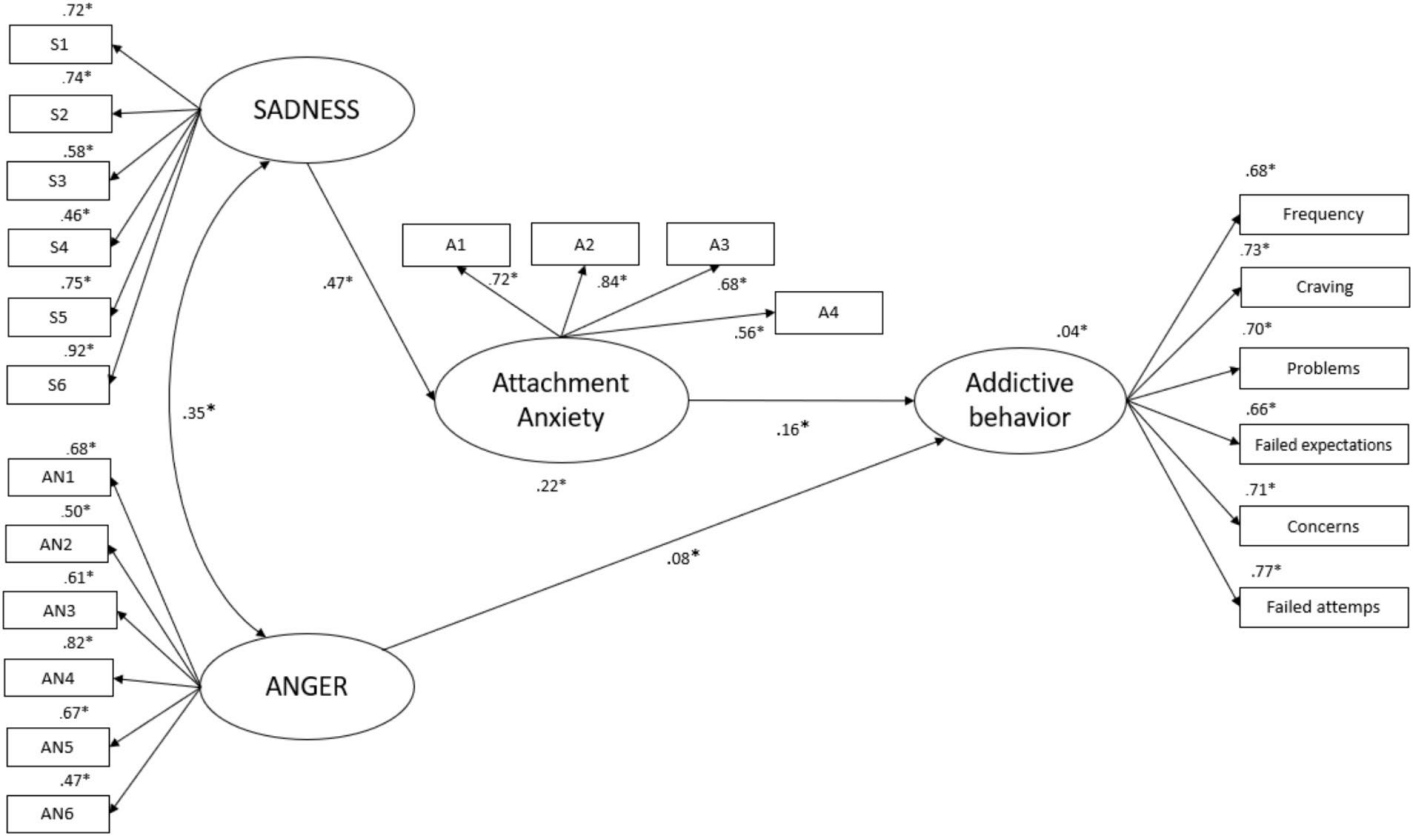
Depiction of the final mediation model of primary emotions SADNESS and ANGER, addictive behavior and attachment anxiety as a mediator variable. ^*^p < 0.001; curved arrow indicates significant correlations (*p* < 0.001). The model was corrected for the effects of sex.

This approach resulted in a model with an acceptable fit to the data: RMSEA = 0.046 (90% CI: 0.041, 0.050); TLI = 0.943; CFI = 0.952; AIC = 784.916. According to the pruning scheme, the model was trimmed by deleting all non-significant paths between variables. This involved: (a) the path between ANGER and attachment anxiety; and (b) the path between SADNESS and addictive behavior. The final standardized solution for the structural equation model is illustrated in Figure 2. The standardized solution for the structural equation model showed an excellent fit: RMSEA = 0.045 (90% CI: 0.041, 0.050); TLI = 0.944; CFI = 0.952; AIC = 782.124. Compared to the initially hypothesized model, the AIC of the final model was smaller, the reduction in AIC score was ∆ 2.79, suggesting that the final model was more parsimonious than the initial model. Overall, this model was able to explain 4% of the variance of addictive behaviors and 22% of the variance in attachment anxiety.

#### Direct effects

As shown in Figure 2, in this structural equation model, there was a significant unique effect from primary emotion ANGER to addictive behavior symptoms (*β* = 0.08, BCa 95% CI [0.02, 0.15]), but not to attachment anxiety (*p* > 0.05) and ANGER was significantly correlated with the primary emotion SADNESS (*r* = 0.35; *p* < 0.001). In addition, there was a direct effect of primary emotion SADNESS to anxious attachment (*β* = 0.47, BCa 95% CI [0.41, 0.53]). Moreover, there was an effect of anxious attachment to addictive behavior symptoms (*β* = 0.16, BCa 95% CI [0.10, 0.22]) but no direct significant effect from primary emotion SADNESS to addictive behavior (*p* > 0.05).

#### Indirect effects

Furthermore, a full mediated significant indirect effect of SADNESS on addictive behavior (*β* = 0.075, Percentile bootstrap 95% CI [0.05, 0.11]) was observed. Its indirect effect on addictive behavior was mediated through its association with anxiety attachment.

## Discussion

This study investigated the mediating role of attachment anxiety on the effect of primary emotions and substance related addictive behavior. In general, our results suggest, as that the link between symptoms of addictive behavior and primary emotion SADNESS was fully mediated by attachment anxiety, while ANGER had a direct effect.

At large, the association between primary emotion SADNESS and ANGER and addictive behavior are echoing previous results: Unterrainer et al. (22) found increased SADNESS, FEAR, and ANGER disposition in patients with substance use disorders. In this study, FEAR was not independently associated with addictive behavior, which is in line with Fuchshuber et al. (23), who showed that substance use was associated with SADNESS and ANGER. Yet, considering the relatively small percentage of total variance explained, tendency toward addictive behavior may be less related to primary emotions dispositions than previously assumed. This is especially the case for SEEKING, which showed no significant associations with addictive symptoms, in agreement with Unterrainer et al. (22). This finding, which is in contradiction with assumptions from theoretical and experimental neuroscientific research (16–18), could be related to conceptual differences between functional aspects of the ML-DA or SEEKING system and the overall disposition for SEEKING measured by the (B)ANPS. Specifically, the ML-DA/SEEKING network seems to be essential for the development of the SUD regarding its involvement in reinforcement learning. However, this may not be reflected in an individual’s predisposition to reduced SEEKING. In general, it has to be emphasized that the psychometric assessment of primary affect dispositions necessarily relies on their tertiary process based symbolic representations (50, 51) and, hence, provides only indirect measures of the underlying concepts.

An alternative explanation might be that current substance consumption patterns of participants could have artificially increased the individual’s SEEKING tendency at the time of the study (14). Clinical studies will be needed to further investigate this issue.

However, the results showed significant indirect and direct effects of the negative primary emotions SADNESS and ANGER on addictive behavior, which is partially consistent with AN assumptions emphasizing an influence of dysregulation within the predominantly opioid-controlled SADNESS system (14, 16, 52), and additionally support observations of object relations theory, emphasizing the role of aggression in substance use disorders (20, 21).

In addition, these results reaffirm the idea of substance use as a method of artificial affect regulation (53). Individuals struggling with addictive behavior may turn to drugs as a way to fill gaps within a disrupted personality structure, which is connected to increased negative affects (8, 53). Specifically, addictive behaviors appear to be connected to increased sensations of loneliness and isolation, as well as intensified feelings of rage and aggression, which all are highly unpleasurable for individuals.

As hypothesized, anxious attachment was significantly associated with tendency toward addictive behavior and with both SADNESS and ANGER. Our findings are not only consistent with the conceptualization of anxious attachment as an hyperactivation strategy, but also with the assumptions of Liese et al. (39), who hypothesizes that insecure attachment, in particular anxious attachment and related emotion regulation deficits, might be a psychological characteristics that increase risk for substance related addictions. Moreover, previous research has observed considerable heritability of both adult attachment (42) and primary affect dispositions (54) and highlighted links between insecure attachment and candidate genes related to oxytocin and mu-opioid receptors (42, 43). Hence, a next research step might be the investigation of shared genetic markers for both adult attachment and primary affect dispositions, which could serve as the biological foundation of the psychometrical associations observed in the present and previous studies (44).

The connection between tendency toward addictive behavior and SADNESS underlines the understanding of addiction as an attachment disorder that is particularly linked to dysregulations in the endogenous opioid system (26, 27). Correspondingly, Zellner et al. (14) described the attachment bond as the prototype of the first addiction. The construct of the activated primary emotion SADNESS can be understood, in the sense of attachment distress or mental pain when being abandoned. The completely mediated association between SADNESS and the tendency toward addictive behavior may result because the attachment disposition may serve for affect regulation and thus regulate SADNESS/separation pain. In addition, the connection between tendency toward addiction and ANGER could be interpreted as support for psychoanalytic theories that connect substance use to auto-aggressive behavior directed against representations of malicious objects and the inner self (19–21).

What is more, the results imply a differential role of primary emotions in the development of psychopathology. Thus, SADNESS may be an essential factor in a broad range of psychiatric disorders (55–57). In accordance to this, the present connection between SADNESS and attachment anxiety symptoms could be associated with results of the meta-analysis by Kossowsky et al. (58), regarding the connection between SADNESS and anxiety disorder symptoms. The authors concluded that the risk of anxiety disorders in adulthood is influenced by the separation anxiety disorder experiences in childhood, which could lead to an insecure attachment style. Regarding Alcaro and Panksepp’s (16) conceptualization of the neuroarchitectural structure of the SADNESS systems, the connection between SADNESS and anxiety disorders symptoms might be attributed to a similar neurological substrate (59).

However, we observed that self-rated primary emotion dispositions and attachment anxiety explained only a small portion of the variance in symptoms of addictive behavior: The present study was able to explain 4% of the variance from the tendency to engage in addictive behaviors. This weak variance explanation may be due to the nature of the sample: It is important to reemphasize that the present sample is not a clinical one. In fact, there were fewer participants in the survey who used substances or used substances to a lesser degree than expected, especially in regards to Fuchshuber et al. (23). The reduced variability in the addiction variable might have contributed to the rather small associations.

### Limitations

This study examined a recursive model, yet interpretations about the direction of influence between these concepts have to be kept speculative at this point. Future studies could examine more complex models of the affective-cognitive framework and examine non-recursive connections between primary emotions, attachment anxiety and the tendency to addictive behavior.

What is more, the present study assessed substance-related problems by means of the global continuum of substance use risk. Yet, problematic consumption of different substance classes might be related with differential primary emotion dysregulations (14). Consequently, future investigations should examine the affective profiles for specific substance related issues. In this context, it would be of further interest to examine whether attachment styles act as a mediator between primary emotions and non-substance addictions (for example, smartphone addiction, etc.).

A further limitation is that self-report measures were employed that reflect consciously available representations of primary emotion dispositions and attachment organization, whereas internalized attachment representation are hypothesized to be at least partly unconscious (60). Additionally, primary affects are often conceptualized as anoetically conscious (7), yet – as discussed above – their assessment via psychometric personality inventories are necessarily indirect, as the measurement depends on language and memory based tertiary process representations.

Along these lines, in our assessment other psychopathologies than substance use risk were only assessed via a single self-rated question regarding a current psychiatric diagnosis. Answers based upon this assessment have only limited descriptive value and hence, do not possess sufficient reliability to be used in further analysis. Future studies should assess comorbid psychopathology in a more detailed manner.

Another restriction of this study is the components of the used sample. Even though the authors tried to ensure diversity, the majority of participants were female young adults, which might be in partly related to the fact that the survey was advertised and administered online. Another reason for the composition of the sample, that the survey was distributed over the networks from German and Austrian universities. So it’s not surprising to see, that the majority of participants are healthy students from Austria and Germany. With regard to the effect of sex, we did not observe significant differences between male and female participants. While in general, substance related addictive behaviors have a higher prevalence in men (61), especially with regard to studies using self-rating instruments, this relation might be difficult to assess, as substance use is more socially stigmatized for women, which in turn might make it more difficult to gather precise information on this matter.

Moreover, as there is evidence that attachment and primary emotion dispositions are interwoven with the biography of individuals (44, 62), future studies should consider gathering information regarding childhood experiences and parental styles.

To be able to make more significant statements about the associations between primary emotions and the tendency toward addictive behavior, as well as the mediating role of attachment anxiety, it would be reasonable to examine these research aims in a clinical population. Based on these limitations, the results of the present study should be seen as supporting evidence which might help to guide future research in clinical populations.

## Conclusion

The results suggest SADNESS, ANGER as well as attachment anxiety as significant risk factors for addictive behaviors. This underscores the importance of considering attachment styles and primary emotions regarding treatment and prevention of addictive behavior. In summary, the findings contribute to a better understanding of how attachment anxiety, primary emotions SADNESS and ANGER and addictive behavior might be related and suggest a focus on attachment styles in a psychotherapeutic setting may be a good way to promote adaptive self-regulation of emotions to reduce the use of maladaptive compensation strategies.

## Data availability statement

The raw data supporting the conclusions of this article will be made available by the authors, without undue reservation.

## Ethics statement

The studies involving humans were approved by Ethics committee of the University of Graz. The studies were conducted in accordance with the local legislation and institutional requirements. The participants provided their written informed consent to participate in this study.

## Author contributions

JF: Writing – review & editing. DA: Writing – original draft. TP: Writing – review & editing. LR: Writing – review & editing. BS: Writing – review & editing. AS: Writing – review & editing. H-FU: Writing – review & editing.
